# Chromosome-Level Genome Assembly of the Yeast Candida verbasci

**DOI:** 10.1128/mra.00005-23

**Published:** 2023-02-22

**Authors:** Broňa Brejová, Viktória Hodorová, Hana Lichancová, Eunika Peričková, Veronika Anna Šoucová, Matthias Sipiczki, Tomáš Vinař, Jozef Nosek

**Affiliations:** a Department of Computer Science, Faculty of Mathematics, Physics and Informatics, Comenius University in Bratislava, Bratislava, Slovak Republic; b Department of Biochemistry, Faculty of Natural Sciences, Comenius University in Bratislava, Bratislava, Slovak Republic; c Department of Genetics and Applied Microbiology, University of Debrecen, Debrecen, Hungary; d Department of Applied Informatics, Faculty of Mathematics, Physics and Informatics, Comenius University in Bratislava, Bratislava, Slovak Republic; University of California, Riverside

## Abstract

Candida verbasci is an anamorphic ascomycetous yeast. We report the genome sequence of its type strain, 11-1055 (CBS 12699). The nuclear genome assembly consists of seven chromosome-sized contigs with a total size of 12.1 Mbp and has a relatively low G+C content (28.1%).

## ANNOUNCEMENT

Candida verbasci is classified into the clade *Lodderomyces/Candida* of the family *Debaryomycetaceae* (subphylum *Saccharomycotina*), which includes important human pathogens (e.g., Candida albicans, Candida parapsilosis), along with the yeasts isolated from various insects (e.g., Candida corydali) (1–4). *C. verbasci* was originally identified in a microbial community associated with *Verbascum* flowers in Tbilisi, Georgia. As this yeast is not osmotolerant, it has been suggested that its presence in flowers resulted from insect transmission rather than natural propagation in sugar-rich nectar (1).

We analyzed the DNA of the type strain, 11-1055, using a combination of long- and short-read sequencing technologies. Total cellular DNA was isolated using a standard protocol (5) from an overnight culture grown in yeast extract-peptone-dextrose (YPD) medium (1% [wt/vol] yeast extract, 2% [wt/vol] peptone, 2% [wt/vol] glucose) at 28°C. In total, 1.9 Gbp (~157× coverage) was obtained in 197,400 long reads (mean, 9,744.8 nucleotides [nt]; longest read, 145,767 nt; *N*_50_, 18,226 nt) using a MinION Mk1B device with an R9.4.1 flow cell and a rapid barcoding sequencing kit (SQK-RBK004; Oxford Nanopore Technologies). A paired-end (2 × 151-nt) TruSeq PCR-free DNA library was also sequenced using the NovaSeq 6000 platform at Macrogen (South Korea), yielding 82,821,710 short reads (12.5 Gbp; coverage, ~1,028×).

The genome sequence was assembled from Nanopore data using Flye v.2.8.3-b1695 (6). The assembly consisted of seven contigs and one scaffold. The only gap in the scaffold, immediately following the rDNA cluster, was filled manually with the corresponding 250-bp portion of an assembly created using miniasm v.0.3-r179 (7), with read overlaps found using Minimap v.2.13-r852-dirty (8), and polished using two iterations of Racon v.1.3.1 (9). The entire assembly was polished using three iterations of Pilon v.1.21 (10), with Illumina reads aligned using BWA-MEM v.0.7.17-r1188 (11), with a single rDNA repeat polished separately and used to replace copies in the assembly to avoid problems with ambiguous mapping of reads.

**FIG 1:**
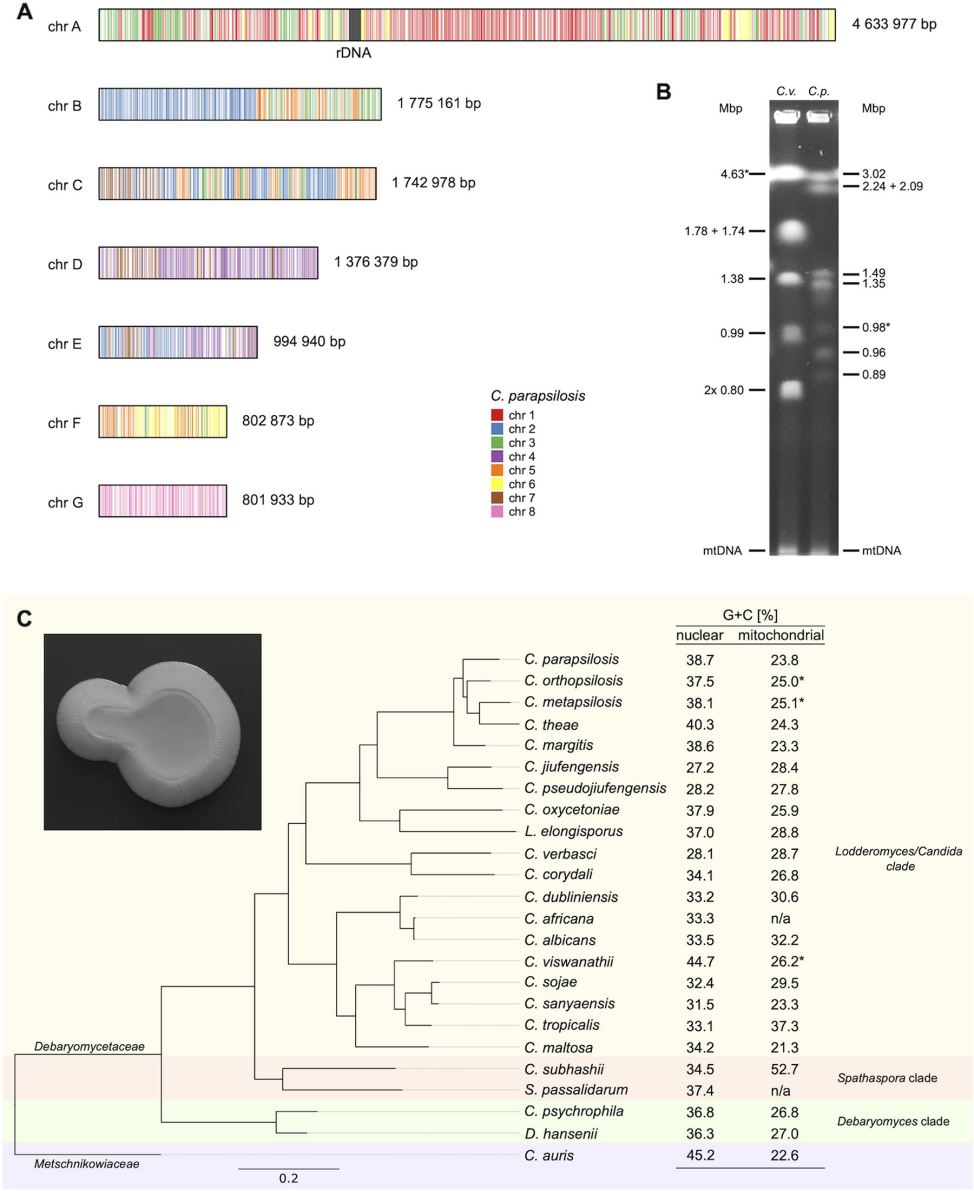
(A) Nuclear chromosomes of *C. verbasci*, colored based on their homology with C. parapsilosis strain CLIB214 (17). Local alignments between the two genomes were computed using LAST v.830 (lastal with option -E1e-10, followed by last-split) (18) and visualized using the ggplot2 v.3.3.6 library in R (19). (B) Electrophoretic karyotype of *C. verbasci* (*C.v.*), analyzed using a CHEF Mapper XA Chiller system (Bio-Rad), with a 120° angle between the electric fields, at the following settings: 120-s pulses for 20 h, followed by 240-s pulses for 28 h at 4 V/cm in a 0.8% agarose gel and 0.5× TBE buffer (45 mM Tris-borate, 1 mM EDTA) at 14°C. The chromosomes of C. parapsilosis strain CLIB214 (*C.p.*) were used as size markers (for more details, see reference 17). Asterisks indicate the chromosomes containing rDNA repeats. mtDNA, mitochondrial DNA. (C) Phylogenetic tree of the *Lodderomyces* clade species, constructed using the BUSCO_phylogenomics pipeline (20) from 2,120 single-copy orthologs present in at least 50% of the genome assemblies (options: –psc 50 –supermatrix) identified using BUSCO v.5.1.2 (21). The G+C contents of the nuclear and mitochondrial DNAs are shown. Asterisks indicate that the nuclear and mitochondrial DNA sequences were obtained from different strains of the species; n/a indicates that the sequence of mitochondrial DNA is not available. The inset illustrates a colony of *C. verbasci* grown for 7 days on a YPD plate.

The final assembly contains one mitochondrial and seven nuclear contigs, with overall G+C contents of 28.7% and 28.1%, respectively. The nuclear contigs, terminated at both ends with telomeric arrays (CAACAAACACTTGAGGTAAGGATG)_n_, correspond in length to the electrophoretic karyotype (Fig. 1A and B) and likely represent full-length chromosomes. To annotate the protein-coding genes, C. albicans and *C. corydali* proteomes were downloaded from UniProt (12) and Shen et al. (13, 14), respectively, and aligned with the assembly using exonerate v.2.4.0 (15). Protein alignments were used as hints in Augustus v.3.2.3 (16), in the first iteration with parameters for C. albicans and in the second iteration with parameters trained on the predictions matching *Candida* proteins with at least 70% identity. In total, 5,313 protein-coding genes were annotated in the nuclear genome of *C. verbasci*.

The chromosome-level genome assembly of *C. verbasci* will be instrumental in further functional analyses, as well as in comparative studies of pathogenic and nonpathogenic species from the clade *Lodderomyces* (Fig. 1C).

### Data availability.

The genome assembly and Illumina and Nanopore reads have been deposited in the European Nucleotide Archive (ENA) and GenBank under accession numbers CANTUO000000000, ERR10286776, and ERR10286775, respectively. The version described in this paper is the first version, CANTUO010000000. The annotated mitochondrial DNA sequence was also deposited at GenBank (accession number OK589855). The results are also available in the genome browser at http://genome.compbio.fmph.uniba.sk/.
